# Lysyl oxidase (LOX) family proteins in extracellular matrix homeostasis: roles in disease and radiation-induced pathogenesis

**DOI:** 10.1007/s11033-026-12438-x

**Published:** 2026-08-07

**Authors:** Jingxian Yang, Vanessa Weyer, Ahmad Sami, Carsten Sticht, Frank A. Giordano, Carsten Herskind, Marlon R. Veldwijk

**Affiliations:** 1 Cellular and Molecular Radiation Oncology Lab, Department of Radiation Oncology, Medical Faculty Mannheim, Universitätsmedizin Mannheim, Heidelberg University, Mannheim, Germany; 2 DKFZ Hector Cancer Institute at University Medicine Mannheim (M028), Heidelberg, Germany; 3 Core Facility Platform Mannheim (CFPM), Medical Faculty Mannheim, Heidelberg University, Mannheim, Germany; 4 Department of Radiation Oncology, Medical Faculty Mannheim, Universitätsmedizin Mannheim, Heidelberg University, Mannheim, Germany; 5 Mannheim Institute for Intelligent Systems in Medicine (MIISM), Heidelberg University, Mannheim, Germany

**Keywords:** Lysyl oxidase family, Extracellular matrix remodeling, Organ fibrosis, Radiotherapy, Therapeutic target

## Abstract

The extracellular matrix (ECM) not only provides structural support in tissues but also regulates cellular processes and tissue homeostasis. The lysyl oxidase (LOX) family of enzymes, consisting of LOX and lysyl oxidase-like enzymes (LOXL1-4), is involved in ECM remodeling by catalyzing the crosslinking of collagen and elastin. Dysregulation of these enzymes is implicated in the pathogenesis of various diseases, including organ fibrosis and tumor development. Furthermore, LOX family members have been shown to affect radiotherapy responses, with specific involvement in radioresistance and radiation-induced fibrosis. This review summarizes the structural and functional characteristics of the LOX family, highlighting their roles in fibrotic diseases such as pulmonary fibrosis, liver fibrosis, and cardiac diseases, their roles in tumor progression, relevance in radiotherapy, and treatment targeting LOX and LOXLs. Understanding the underlying mechanisms and potential therapies is critical for developing novel strategies in both disease identification and management.

## Introduction

Extracellular matrix (ECM) is a well-organized three-dimensional scaffold that provides physical support for cells and plays essential roles in regulating cellular processes and tissue homeostasis [1]. It consists of diverse structural and functional macromolecules - including fibril-forming proteins like collagens and elastin, adhesive glycoproteins like fibronectin and laminins, non-fibrillar components such as proteoglycans and glycosaminoglycans, and cell surface receptors. Collagen is the most abundant component, accounting for more than 30% of ECM mass and is actively involved in ECM dynamic remodeling of connective tissue [1]. In physiological settings, collagen synthesis, leading to an increased ratio of collagen types III: I, contributes to the recovery of tissue tensile strength. It guides cell adhesion, migration and proliferation, modulates immune responses, and promotes angiogenesis in the four classic phases of wound healing (hemostasis, inflammation, proliferation, and remodeling) [2]. Under pathological conditions, activated fibroblasts (predominantly myofibroblasts) are closely associated with tumor metastasis and tissue fibrosis. These fibroblasts help form the tumor stroma by synthesizing ECM proteins, secreting cytokines, recruiting immune cells and remodeling supportive structures [3]. Excessive collagen deposition, altered alignment methods, along with the high interstitial fluid pressure caused by increased stiffness, collectively facilitate tumor cell dissemination and therapeutic resistance [4]. In chronic wound healing, continuous repair activation leads to the accumulation of fibrillar collagens. After a series of collagen deposition and degradation processes in the ECM, fibrosis is initiated, characterized by structural disorganization and enhanced crosslinking, eventually resulting in scars or organ fibrosis [5].

Cross-linking, a major posttranslational modification of collagen, is one of the key factors underlying these effects. It provides tissue with mechanical stability and enzymatic resilience [6]. There are two cross-linking mechanisms in vivo: enzymatic crosslinking and non-enzymatic crosslinking [7]. Among the former, lysyl oxidase (LOX) has been extensively explored because lysyl oxidase-mediated enzymatic crosslinking plays a central role in controlling the quantity, quality and maturation of collagen fibrils [8]. LOX was first identified in the 1960s as an extracellular enzyme responsible for crosslinking of elastin and collagen [9]. To date, four additional lysyl oxidase-like enzymes (LOXL1-4) with the same C-terminal catalytic domain have been characterized in mammals, together forming the LOX protein family [10]. Besides the catalytic activity, different tissue expression patterns and recently validated intracellular functions of LOX family members have been implicated in the progression of various diseases, particularly in tumor development and fibrotic processes across organs [11, 12]. Therapeutic strategies targeting this enzyme family are currently under active investigation. This review aims to provide an up-to-date overview of the LOX family, with a focus on their physiological functions and pathological roles in fibrosis, cancer, and radiation-induced conditions, highlighting its potential as an effective target for therapeutic intervention.

## Structural and functional characteristics of the LOX family

### Protein structure

**Fig. 1:**
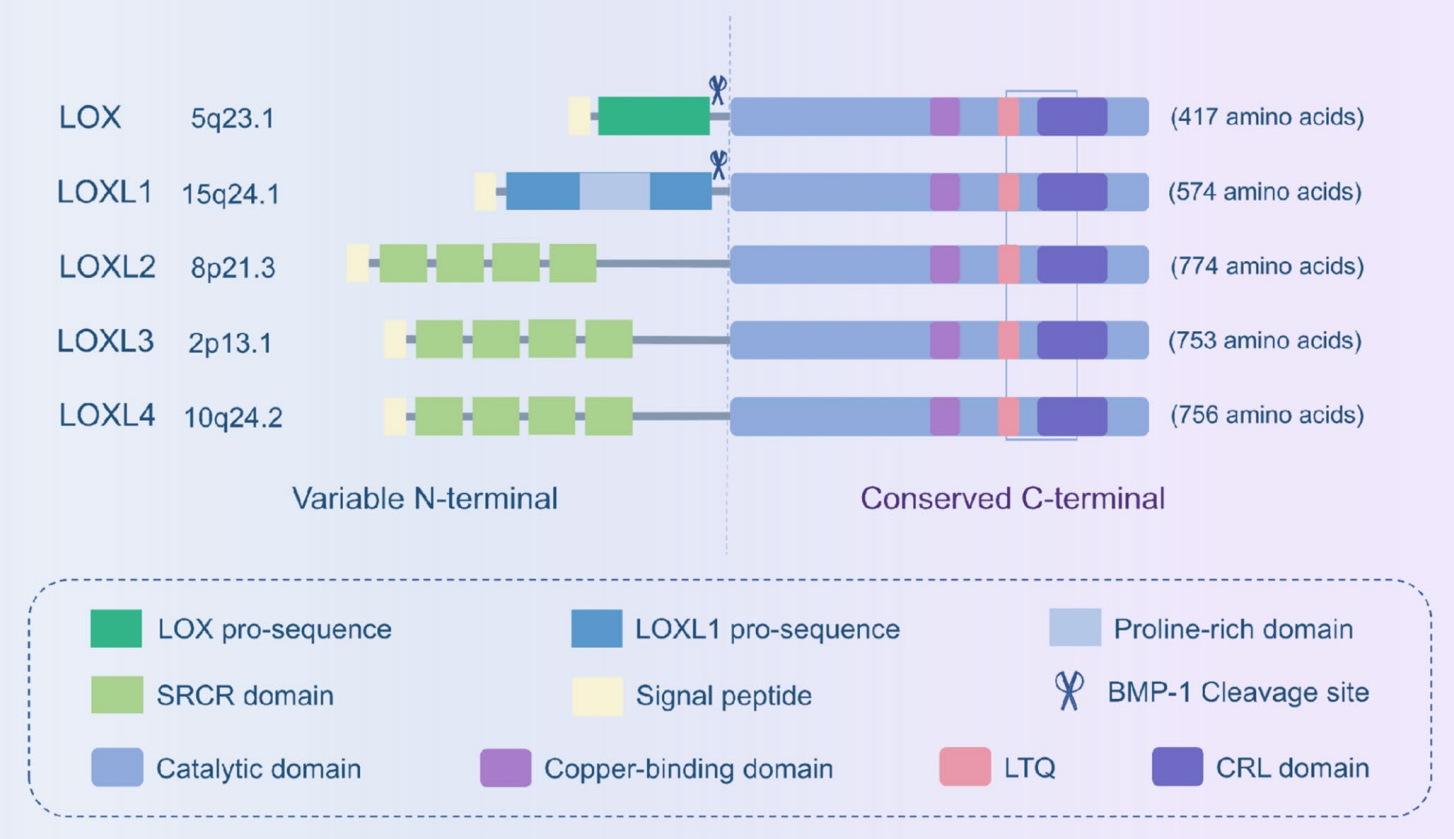
Schematic illustration of the domain structure of LOX family members

LOX enzymes are secreted copper-dependent amine oxidases which are essential for multicellular animals (metazoans) but the LOX gene family can be traced back to unicellular pro- and eukaryotic organisms [10]. In humans, LOX and LOXL1-4 are encoded by homologous genes located on chromosomes 5, 15, 8, 2, and 10, respectively, with a variable number of exons [13]. All share a highly conserved domain at their C-termini, which contains essential motifs for copper binding, lysine tyrosylquinone (LTQ) cofactor, and a cytokine receptor-like (CRL) region [11]. LTQ is a covalently bound cofactor formed by a quinone crosslink between lysine and tyrosine side chains within the LOX molecule. As the catalytic center, the copper ion participates in the catalytic process of LTQ formation, and then LTQ oxidizes lysine residues in collagen or elastin into aldehydes (LysAld), thereby initiating covalent crosslinking between protein molecules [12]. Based on their diverse N-terminal domains, the family members can be divided into two subfamilies: LOX/LOXL1 with prodomains, and LOXL2-4 with four scavenger receptor cysteine-rich (SRCR) domains (Fig. 1). LOX and LOXL1 need to be cleaved into active mature forms by BMP1 (bone morphogenetic protein 1) proteases, whereas LOXL2-4 undergo non-proteolytic functional maturation to exert further enzymatic functions and protein-protein interactions [14].

**Fig. 2:**
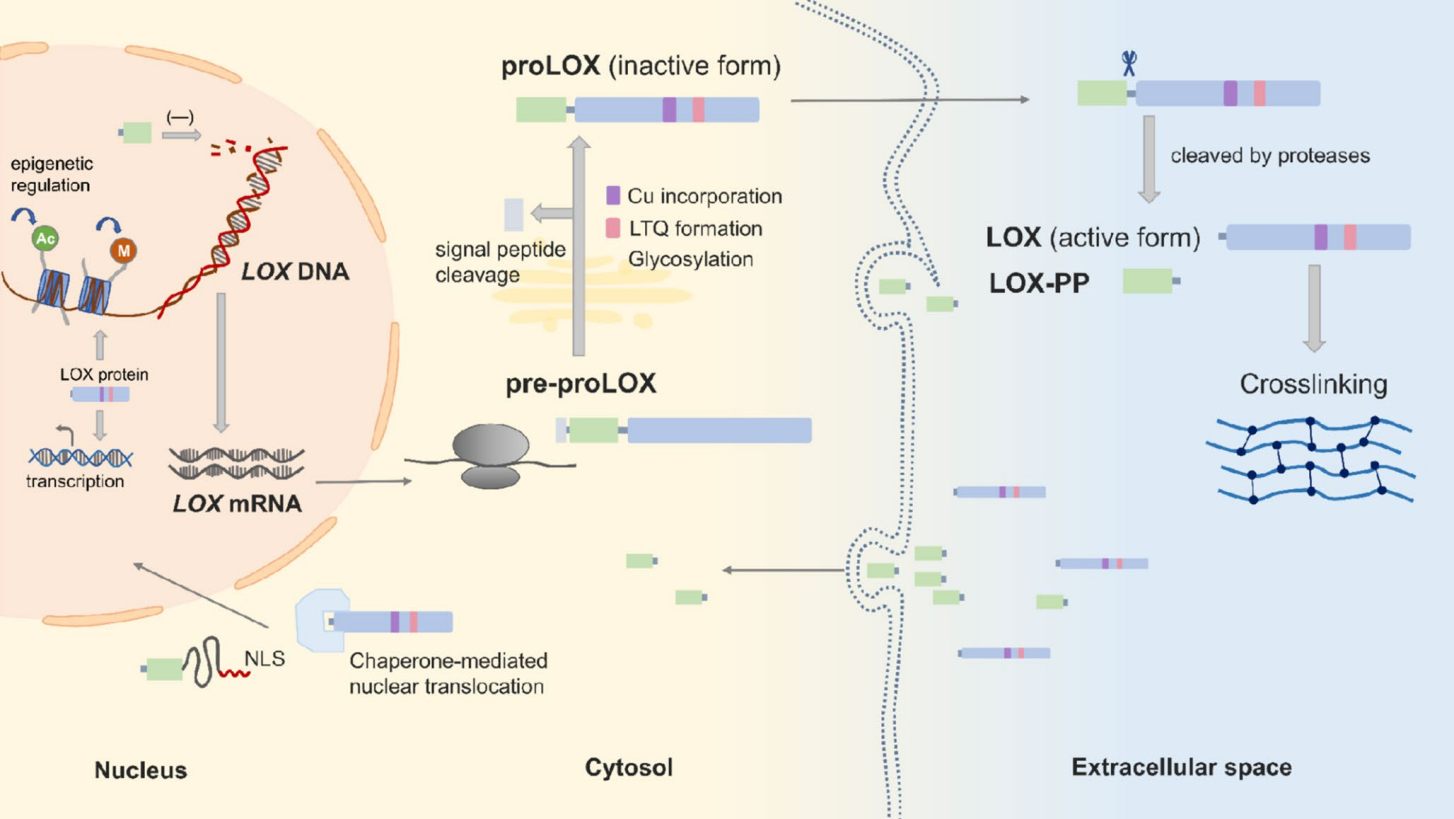
Schematic illustration of cellular LOX production and processing. Modified from Rodríguez C, Martínez-González J (2019) [15].

The expression and function of LOX activity can be regulated at three levels: transcriptional control, proenzyme processing, and direct modulation of enzymatic function (Fig. 2) [15, 16]. Transcription is activated via a number of growth factors and cytokines, including transforming growth factor β1 (TGF-β1), as well as integrins, and hypoxia-inducible factor-1α (HIF-1α) [17]. Gene transcription levels have also been shown to be epigenetically modulated by DNA methylation in promoter regions [18]. The peptides are translated as a proenzyme with a signal peptide (pre-proLOX) which undergoes post-translational modifications (including signal peptide cleavage, copper incorporation, LTQ cofactor formation, and N-glycosylation of the propeptide) in the endoplasmic reticulum (ER) and Golgi apparatus, leading to the generation of the inactive proenzyme (proLOX) with an approximate molecular weight of 50 kDa. Finally, proLOX is secreted into the extracellular space and proteolytically cleaved by proteases to generate the 32 kDa active mature LOX and the 18 kDa LOX propeptide (LOX-PP).

Both the mature LOX protein and LOX-PP have also been shown to translocate into the nucleus. Secreted LOX-PP is primarily internalized by cells through dynamin-independent macropinocytosis and may be directed into the nucleus via its putative nuclear localization signal (NLS), exerting tumor-suppressive and regulatory effects [19, 20]. In the cytoplasm, LOX-PP interacts with multiple binding partners including heat shock protein (Hsp70)/c-Raf, tubulin, fibroblast growth factor receptors (FGFRs), and receptor-type protein tyrosine phosphatase kappa (RPTP-κ). Through these interactions, it inhibits canonical oncogenic signaling cascades, notably MAPK, PI3K/Akt, nuclear factor kappa B (NF-κB), and β-catenin pathways, leading to reduced proliferation and invasion of cancer cells [12, 20]. After nuclear translocation, LOX-PP was found to bind to the MRE11-RAD50-NBS1 complex, the primary sensor of DNA double-strand breaks, thereby impairing DNA repair capacity [21]. For LOX, the mature enzyme secreted into extracellular space can be re-internalized and accumulate in the cell nucleus [22]. Early studies suggested that it contains potential NLS which enable active nuclear import, while subsequent research revealed that LOX lacks any known NLS and may instead undergo partner protein-mediated nuclear translocation [23, 24]. Although the specific mechanism remains unclear, mature LOX in the nucleus was found to modulate chromatin structure and gene expression, thereby participating in tumor development and cell differentiation [25].

### Biological functions

The conserved core function of all LOX family members is to initiate covalent crosslinking in collagen and elastin, thereby promoting ECM maturation and stabilization. The enzymes catalyze the oxidative deamination of specific lysine and hydroxylysine residues in collagen and elastin. The generated reactive aldehydes then spontaneously undergo condensation reactions with neighboring ε-amino groups or with other aldehydes, including Schiff base formation and aldol-type condensations, resulting in the formation of intra- and intermolecular covalent cross-links [20]. Subsequently, trivalent crosslinking will be formed to maintain the tensile strength, insolubility, and structural integrity of collagen and elastin fibers within connective tissues [8].

LOX and LOXL2 are the most widely investigated members of the family with additional characterized biological functions. LOX is essential for embryogenesis and organ development. LOX deficiency in mice results in abnormal development of skin, muscle, respiratory, and cardiovascular systems, and can even lead to perinatal lethality [16]. LOX can also regulate cell adhesion and migration, osteogenic differentiation, angiogenesis and accumulates in the nucleus to mediate oxidative modifications of histones [17, 24]. LOXL2 has both intracellular and extracellular functions, one of the most notable of which is the induction of epithelial-mesenchymal transition (EMT). EMT plays a pivotal role in embryonic development and is characterized by cell adhesion and motility. By modulating Snail-mediated repression of E-cadherin, LOXL2 induces the EMT-related phenotypic switch, contributing to pathological tissue remodeling and disease progression [26, 27]. At the same time, LOXL2 participates in tumor angiogenesis by regulating the endothelial-mesenchymal transition (EndMT) via protein kinase B (PKB)/Akt and focal adhesion kinase (FAK) signaling pathways [28].

LOXL1 is crucial for elastic fiber maintenance and ECM homeostasis. Thus, LOXL1 deficiency has been linked to elastic tissues disorders such as pelvic organ prolapse and ocular hypertension. It is regarded as a promising research direction and therapeutic target for glaucoma, especially exfoliation glaucoma [29]. In addition to its shared involvement in tumor progression and fibrotic diseases, recent research on LOXL3 has identified it as a regulator of cell proliferation and inflammatory responses via the LOXL3–STAT3 axis, and it is also associated with embryo viability and organ development [30]. LOXL4, on the other hand, has primarily been investigated as a prognostic marker in cancers [31]. The expression of the five LOX family members varies across different tissues (Table 1), reflecting not only their functional diversity in maintaining biological functions, but also offering important insights into their roles in the development and progression of diseases.

**Table 1:** Distribution of LOX family members in organs

Family member	mRNA distribution*	Protein distribution
LOX	heart, gallbladder, breast, bladder, lung	n/a
LOXL1	heart, bladder, prostate, uterus, lung	appendix, colon, esophagus, rectum, small intestine, stomach
LOXL2	placenta, colon, ovary, breast, appendix	multi-organs across the nervous, endocrine, respiratory, digestive, urinary, and reproductive systems
LOXL3	placenta, bone marrow, spleen, heart, appendix	duodenum, small intestine, pancreas, placenta
LOXL4	salivary gland, breast, cervix, seminal vesicle, colon	lung, kidney, placenta, breast, spleen, bone marrow

*The organs are listed based on the expression levels from high to low, with the top five. Data sourced from the Human Protein Atlas Consensus dataset (http://www.proteinatlas.org/)

## Role of LOX family enzymes in diseases

Accumulating evidence indicates that the pathological significance of LOX family enzymes extends beyond their structural functions. Sustained activation of ECM crosslinking enzymes can lead to a disorganized and stiffened ECM. The mechanical changes promote fibroblast/myofibroblast activation, thereby driving the self-perpetuating progression of fibrosis [27]. In cancer, the multifaceted roles of LOX family members in facilitating tumor invasion and metastasis are noted. Through both enzymatic and non-enzymatic mechanisms, these enzymes participate in the amplification of signaling pathways, immune cell recruitment, remodeling of the pre-metastatic niche, angiogenesis, and the indirect regulation of nuclear functions [25]. Disease-related stimuli, including oxidative stress, hypoxia, inflammation, and mechanical stress, can induce the expression or activity of the enzymes, establishing a positive feedback loop among pathological signal transduction, fibrotic remodeling, and mechanical reprogramming [32]. Thus, LOX family enzymes are not only canonical downstream effectors, but also active contributors to disease induction and progression by linking the dynamic ECM to the formation of the pathological microenvironment.

### Organ fibrosis

Fibrosis is a pathological process characterized by the excessive deposition of ECM components. It is a result of an aberrant repair response triggered by persistent tissue damage or chronic inflammation, leading to sustained fibroblast and myofibroblast activation [3, 33]. This process can affect almost all organs (including lung, liver, heart kidney, skin and bone marrow), causing structural distortion. As progression is typically irreversible, it ultimately results in organ dysfunction and failure, making it a major cause of patient mortality. The excessive deposition primarily consists of collagen, especially types I and III, which form the key structural components of the tissue. Given the crucial role of LOX/LOXL in collagen crosslinking, numerous studies have highlighted the involvement of LOX and its paralogs in the initiation and progression of fibrosis across various organs.

#### Pulmonary fibrosis

Pulmonary fibrosis refers to a group of chronic, progressive lung diseases that include idiopathic pulmonary fibrosis (IPF) as well as other interstitial lung diseases (ILDs) that exhibit a progressive pulmonary fibrosis (PPF) phenotype. Their common manifestations are compromised gas exchange, restrictive ventilation disorders, and they eventually develop into respiratory failure. The causes of pulmonary fibrosis can be idiopathic or secondary to known factors such as infections, environmental exposures, medical treatments, or allergic conditions [34]. These triggers can induce excessive inflammatory responses and fibrotic remodeling under pre-existing inflammation.

As the most relevant lysyl oxidase, LOXL2 shows consistently increased expression in mouse lung fibrosis, human IPF lung tissues, airway smooth muscle cells, and fibroblast foci [35]. It plays a regulatory role in fibrogenesis through the TGF-β/Smad pathway. The TGF-β superfamily are important cytokines that regulate various biological processes including extracellular matrix synthesis. TGF-β1, is one of the three TGF-β isoforms and is the key activator of physiological repair and collagen accumulation [36]. In a bleomycin (BLM)-induced mouse lung fibrosis model, it was found that LOXL2 can upregulate the phosphorylation of Smad2/3, the expression of Smad4 and Snail in the TGF-β1 signaling pathway, while suppressing the expression of the inhibitory factor Smad7 [37]. In asthma, LOXL2 acts as a downstream effector of TGF-β1, strongly promoting EMT and airway remodeling, and it has been shown that changes in LOXL2 also significantly affect AKT expression and activation [26]. In a silica-induced silicosis model, loss of post-transcriptional regulation acting on the 3’ UTR of LOXL2 mRNA leads to increased LOXL2 expression, which results in ECM deposition mediated by direct binding to collagen and elastin [38]. LOXL2 enhances differentiation into α-smooth muscle actin (α-SMA)-positive activated myofibroblasts and collagen strength under pro-fibrotic conditions, synergizing with the occurrence of fibrosis [38, 39].

LOX is upregulated in most studies of pulmonary fibrosis and also acts as a TGF-β1 regulatory protein [40]. In human fetal lung fibroblast cells, BLM-induced elevation in copper concentration leads to the upregulation of LOX activity, which may contribute to the mechanism of this kind of pulmonary fibrosis [41]. In an animal model, BLM-induced injury leads to an increase in copper ion concentration in the lung tissue. This copper dysregulation may be related to a novel form of cell death “cuproptosis” and upregulates LOX activity, thereby aggravating the development of fibrosis [42]. In the inflammatory stage, down-regulation of LOX expression or activity in vivo can significantly decrease the level of TGF-β pathway-related molecules and the number of α-SMA-positive myofibroblasts, thereby alleviating the progression of fibrosis [43, 44].

Unlike LOX and LOXL2, LOXL1 is the only member whose expression is elevated in current smokers and chronic obstructive pulmonary disease (COPD) patients, suggesting its involvement in both the regulation of collagen crosslinking in small airways and the response to external stimuli that modulate collagen crosslinking [45]. The heterogeneity of its expression in IPF also suggests the potential of LOXL1 as a target for combination therapy [40]. LOXL3 and LOXL4 have not been the focus due to their insignificant expression changes in pulmonary fibrosis-related diseases. However, recent perspectives suggest that LOXL3 may play an important role in the fibroblast-to-myofibroblast transition (FMT), while LOXL4 is considered the main driver of pathological collagen crosslinking and the formation of a fibrotic microenvironment [35, 46].

#### Liver fibrosis

Liver fibrosis represents a common pathological consequence of chronic liver injuries and serves as a critical precursor to cirrhosis and hepatocellular carcinoma. Hepatic stellate cell (HSC) has been identified as the central driver for liver fibrogenesis by transdifferentiation into activated myofibroblast-like cells which exhibit proliferative, contractile, inflammatory, and chemotactic properties [47]. The activation of HSC is finely regulated by a complex network of intracellular and extracellular cues. TGF-β signaling is the core fibrogenic pathway, together with platelet-derived growth factor (PDGF) and connective tissue growth factor (CTGF), processing the HSC activation and ECM production. Developmental pathways such as Hedgehog and Notch, extracellular matrix remodeling via integrins, oxidative stress, nuclear receptor signaling, inflammatory reaction and epigenetic mechanisms also contribute to shaping the transcriptional landscape of HSC and sustaining its activated phenotype [48]. These intricate signaling pathways provide a foundation for developing targeted antifibrotic therapies.

LOX, LOXL1, and LOXL2 show aberrant expression and enhanced activity in liver fibrosis when compared to healthy livers, prompting their investigation as potential targets. In the carbon tetrachloride (CCl_4_)-induced rodent liver fibrosis models, Perepelyuk et al. observed that LOX is primarily produced by HSCs and portal fibroblasts in the early stage, mediating the mature trivalent crosslinking to provide matrix stiffness. The increased mechanical tension, in turn, promotes the differentiation of HSCs into myofibroblasts [49]. In the advanced stage of fibrosis progression, Liu et al. demonstrated that LOX activity is essential for the matrix stabilization, as inhibition of LOX increased scar-associated macrophage infiltration, weakened HSC activation, and reduced the irreversibility [50]. Furthermore, it is suggested that hepatocytes are one of the sources of LOX production. The orphan nuclear receptor ERRγ can activate the LOX promoter induced by interleukin 6 (IL-6) in hepatocytes, leading to elevated LOX expression [51]. LOXL1, which is synergistically upregulated with LOX, has recently been recognized as the dominant player in the coregulation loop of HSCs. It enhances the cellular transition signal through the Notch signaling pathway, accelerating the progression of fibrosis [52]. LOXL1 inhibition can not only reduce elastin crosslinking and limit HSC activation, but also attenuate cholestatic liver fibrosis via PDGFRβ/PI3K and ERK signaling [53]. LOXL2, apart from maintaining matrix stabilization, helps promote fibrosis progression by regulating the lineage differentiation of hepatic progenitor cells (HPCs) [54]. Current developments of therapeutic approaches to targeting LOXL2 include natural compounds, miRNA-based epigenetic regulation, and strategies for combining different delivery technologies [55, 56]. In these studies, LOXL2 inhibition also downregulates HSC activity by remodeling ECM. Thus, Cheng et al. highlighted that LOXL2 is a direct downstream target gene of Yes-associated protein (YAP), which positively regulates LOXL2 transcription in HSCs [57]. The roles of LOXL3 and LOXL4 in liver fibrosis remain to be explored.

#### Cardiac diseases

Fibroblasts are the second most abundant cell type in the heart, after cardiomyocytes [15]. They participate in the remodeling of cardiac ECM induced by external stimuli, including mechanical load, ischemic injury, inflammation, and oxidative stress, as well as internal factors such as genetic and metabolic disorders. LOX family members are key enzymes involved in the adverse accumulation of fibrillar collagen matrix and insoluble collagen production [58]. The pathological process of fibrosis and ECM stiffness would impair myocardial diastolic function in ventricle, further disrupting left ventricular function and leading to adverse outcomes such as heart failure. In the atrium, it impedes the propagation of cardiac impulses, resulting in arrhythmias [59]. Both are closely related to the renin-angiotensin-aldosterone system, which is an upstream signaling pathway of TGF-β1 in promoting fibrosis.

LOX, LOXL1, and LOXL3 are highly expressed in the cardiovascular system under physiological conditions, with LOX being the most abundant and functionally significant. A study in a rat model of chronic heart failure revealed that the TGF-β1/Smad2/3 signaling pathway can increase the expression of a key subunit of the transcription factor activator protein 1 (AP-1), thereby upregulating transcriptional induction of LOX [60]. A myocardial infarction model showed that ERK1/2 MAPK signaling is regulated by periostin, an important member of the matrix cell protein family, which can then transcriptionally upregulate serum response factor and promote the enhanced LOX in angiotensin II (Ang II)-stimulated cardiac fibroblasts [61]. Conversely, LOX overexpression in transgenic mouse models results in excessive production of reactive oxygen species (ROS), activating p38 MAPK, thereby exacerbating Ang II-mediated cardiac hypertrophy [62]. In addition, integrin α2β1, osteopontin and Rho-associated kinases (ROCK1 and ROCK2) are considered new factors involved in regulating LOX [15]. For the rest of the family members, although a small amount of data indicates some relevance to cardiovascular dysfunction, their regulation in the pathophysiology of heart diseases remains unclear. LOXL1 may be associated with cardiomyocyte hypertrophy [63]. LOXL2 mediates the crosslinking of cardiac interstitial collagen fibers, and its serum levels can serve as an indicator of atrial fibrosis [64]. LOXL3 is related to vascular wall integrity and can mimic the pro-fibrotic effect of TGF-β1, promoting cardiac fibrosis through the activation of the mechanistic target of rapamycin (mTOR) signaling pathway [65, 66].

#### Other diseases

Renal fibrosis is a common pathological process and ultimate outcome of chronic kidney disease (CKD). Biopsy is the only gold standard for its diagnosis [67]. Combined with clinical and laboratory data analysis, it has been observed that both serum and tissue levels of LOX are significantly elevated and correlated with the severity of renal fibrosis, suggesting that LOX could serve as a biomarker for non-invasive diagnosis of renal fibrosis and clinical patient stratification [68]. It was further found that LOX expression is regulated by the β-arrestin/ERK/STAT3 pathway and then drives excessive collagen crosslinking in ECM during renal fibrogenesis [69]. In the diabetic nephropathy model, LOX expression was induced by TGF-β, leading to the stabilization of Snail protein and promoting partial EMT. This result highlighted the synergistic effect of EMT in tubulointerstitial fibrosis, a core process of renal fibrosis [70]. Similarly, LOXL2 was found to be secreted through exosomes under the induction of TGF-β1, promoting EMT and participating in tubulointerstitial fibrosis [71]. The use of LOX/LOXL2 inhibition can effectively ameliorate renal fibrosis.

LOX has also emerged as a novel therapeutic target in rare fibrotic diseases. Systemic sclerosis (SSc) is an autoimmune disease characterized by widespread fibroproliferative changes and vasculopathy. Skin fibrosis is one of its most prominent clinical features. Although the correlation between LOX levels and the modified Rodnan skin score (mRSS) remains inconclusive, elevated serum LOX levels have been consistently observed in SSc patients [72]. On the one hand, TGF-β1 signaling promotes LOX/LOXL-mediated collagen crosslinking and increases ECM stiffness during the progression of skin fibrosis [73]. In particular, LOXL4 may serve as an important mediator of skin fibrosis, which is induced by TGF-β and regulated through AP-1 [74]. On the other hand, LOX exerts crosslinking-independent pathogenic effects in SSc-related fibrosis. Thus, LOX promotes IL-6 production by inducing c-Fos expression, facilitating its nuclear translocation, and enhancing its binding to the IL-6 promoter [75]. IL-6 is secreted by dermal fibroblasts and directly drives fibrogenesis [76].

**Fig. 3:**
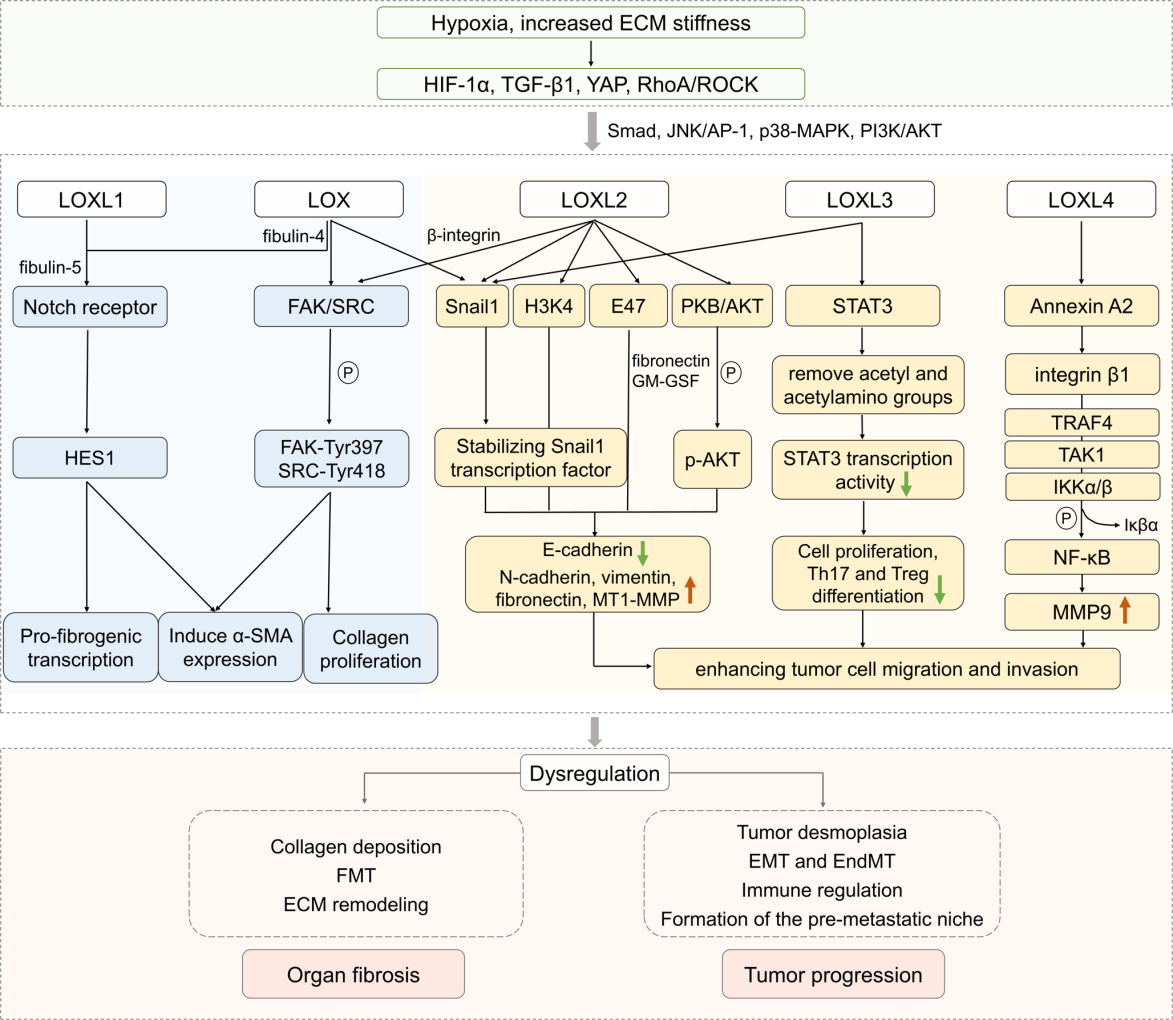
Signaling pathways involving LOX family members in fibrotic diseases and tumor progression. Green arrows: downregulation or inhibition. Red arrows: upregulation or promotion. Circled “P”: phosphorylation. Abbreviation: hairy and enhancer of split 1 (HES1), focal adhesion kinase (FAK), protein kinase B (PKB), granulocyte-monocyte colony stimulating factor (GM-CSF), membrane type 1 matrix metalloproteinase (MT1-MMP), regulatory T cell (Treg), TNF receptor-associated factor 4 (TRAF4), TGF-β activated kinase 1 (TAK1), Iκβ kinase kinase (IKKα/β), nuclear factor-κB (NF-κB), matrix metalloprotease 9 (MMP9), fibroblast-to-myofibroblast transition (FMT), epithelial-mesenchymal transition (EMT), endothelial-mesenchymal transition (EndMT), H3K4: trimethylation of histone H3 at lysine 4 (H3K4me3) in the E-cadherin promoter, E47: a bHLH transcription factor encoded by E2A gene

In primary myelofibrosis (PMF), not only is serum LOX elevated, but all LOX family members are overexpressed, which are undetectable in normal bone marrow [77]. Among them, the activity of LOXL2 has been regarded as an independent adverse prognostic factor [78]. PMF, along with secondary myelofibrosis, belongs to the group of myeloproliferative neoplasms (MPNs) and is characterized predominantly by progressive bone marrow fibrosis. A hallmark of myelofibrosis is dysregulation of the JAK/STAT signaling pathway, leading to pathological ECM remodeling [79]. Within the bone marrow microenvironment, abnormal proliferation and deregulated ploidy of the megakaryocyte lineage have been observed. Studies have shown that LOX is markedly upregulated in megakaryocytes from both transcription factor-deficient mice and transgenic mice carrying a genetic mutation, with enhanced adhesion to collagen [80]. LOX may further potentiate megakaryocyte proliferation by mediating a chemotactic response to PDGF-BB, a dimeric isoform of the PDGF receptor family that activates downstream proliferative signaling [81]. Taken together, LOX/LOXL proteins contribute to organ fibrosis through both enzymatic crosslinking and non-enzymatic cellular regulatory mechanisms, making them multi-dimensional therapeutic targets in fibrotic diseases (Fig. 3).

### Tumor progression

The *LOX* gene was initially named *ras* rescision gene (rrg) as it inhibits *ras* oncogene-mediated cellular transformation, though subsequent studies revealed that the tumor-suppressive effect was attributed to its propeptide LOX-PP [12]. To date, the mature LOX enzyme and its homologs, LOXL1-4, are mostly recognized as pro-tumorigenic factors in various cancers. Their tumor-promoting mechanisms primarily involve tumor microenvironment (TME) remodeling, including tumor desmoplasia, immune regulation, hypoxia and angiogenesis.

In the TME, members of the LOX family are primarily secreted by cancer-associated fibroblasts (CAFs) and tumor cells under hypoxic conditions [82]. Through their enzymatic activity, they promote abnormal collagen deposition and thereby increase matrix stiffness. The mechanical alterations contribute to tumor progression by enhancing cancer cell invasiveness and facilitating metastatic dissemination [83]. As a mechanosensitive signaling molecule, FAK can be activated by hydrogen peroxide (H₂O₂), a byproduct of LOX/LOXL-mediated oxidative deamination. The elevated H₂O₂ levels promote the phosphorylation and activation of the FAK/Src signaling pathway, modulating cell-ECM adhesion and enhancing tumor cell migration [84]. LOX/LOXL also play an active role in EMT, enabling tumor cells to migrate and invade surrounding tissues. In oral squamous cell carcinoma, LOX activates YAP signaling via the non-canonical Hippo mechanotransduction pathway [85]. In gastric cancer (GC), LOX has been shown to activate the TGF-β/insulin-like growth factor 1 (IGF1) signaling pathway, while LOXL1 may induce EMT by upregulating the EMT-associated transcription factor Snail2 [86]. In breast cancer, LOXL1 and LOXL4 are transcriptionally regulated by another EMT core transcription factor ZEB1, thereby promoting invasive behavior [87]. LOXL3 interacts with EMT-inducing transcription factor Snail and contributes to EMT phenotypic switching [88]. LOXL2 is also involved in EMT induction across multiple tumor types, including those of the thoracic, gynecological, and gastrointestinal systems [25].

In addition, the LOX family act synergistically with HIF-1α and matrix metalloproteinases (MMPs) to promote the formation of the pre-metastatic niche [89]. In most solid tumors, tumor cells adapt to hypoxia through the hypoxia-inducible factor signaling pathway. Its activation will result in metabolic reprogramming, angiogenesis, immune evasion, and therapeutic resistance [90]. In glucose metabolism, cancer cells prefer aerobic glycolysis (Warburg effect) to support rapid proliferation. LOX and LOXL2 are reported to stimulate cancer cell proliferation in a HIF-1α–dependent manner: LOX promotes GC cell proliferation by enhancing the Warburg effect via AKT-p70S6K-HIF1-α pathway [91]; LOXL2 stabilizes HIF-1α by inhibiting prolyl hydroxylases and then promotes aerobic glycolysis, facilitating pancreatic cancer cell invasion [92]. Tumor angiogenesis is crucial for the development and progression of tumors. LOX promotes angiogenesis by regulating the expression of vascular endothelial growth factor (VEGF), while LOXL1 interacts with αvβ3 integrin to modulate signaling pathways, directly regulating vascular endothelial cells [93, 94]. LOX and LOXL2 can also mediate vasculogenic mimicry, an endothelial-independent mechanism of blood supply [95, 96]. At the same time, all LOX family members are involved in the regulation of immune cell infiltration, with a focus on enhancing tumor-associated macrophage (TAM) infiltration and restricting T cell infiltration and differentiation [97]. LOXL2 is positively correlated with stemness, while LOXL4 promotes immune evasion by upregulating the expression of the programmed death-ligand 1 (PD-L1) [98, 99]. They then recruit CD11b^+^ bone marrow-derived cells (BMDCs) upon hypoxic induction and, together with MMPs that degrade the ECM, enhance metastatic efficiency [100, 101]. The previously less studied LOXL3 has recently been found to promote tumor progression and chemotherapy resistance by inhibiting ferroptosis [102].

## Role of LOX family enzymes in radiotherapy

Radiotherapy (RT) is one of the cornerstones of cancer treatment, with approximately 50% of all cancer patients treated with curative intent receiving RT as part of the treatment. Various highly conformal treatment methods such as intensity-modulated radiotherapy (IMRT), stereotactic radiotherapy (SBRT), proton therapy, and brachytherapy, have been continuously developed to maximize radiation dose delivery to tumors while sparing healthy tissues from toxicity [103]. Tumor radioresistance, patient treatment tolerance, and management of radiotherapy side effects are also key factors that determine both treatment efficacy and patients' quality of life.

### Radioresistance and IR-induced migratory phenotype

Ionizing radiation (IR) was shown to induce LOX secretion in a dose-dependent manner across multiple tumor cell lines [104]. The altered expression levels of LOX family members are associated with radiotherapy response and tumor cell migratory behavior.

Based on studies on A549 lung adenocarcinoma cells, Shen et al. proposed that the IR-induced LOX secretion is regulated via post-transcriptional mechanisms rather than through increased gene transcription, which is distinct from that under hypoxic conditions. This elevation of LOX can specifically enhance the invasiveness of naïve A549 tumor cells [104]. Gong et al. found that LOX expression was significantly upregulated at both mRNA and protein levels in response to hypoxia and subsequent experiments indicated that LOX may contribute to tumor radioresistance via an enhanced DNA damage response by promoting DNA repair, inducing G2/M cell cycle arrest, and reducing apoptosis [105]. Similarly, Ko et al. demonstrated that HIF-1α expression in radioresistance triple negative breast cancer (TNBC) MDA-MB-231 cells was correlated with LOX secretion [106]. By activating the HIF-1α–LOX axis, a pre-metastatic niche was formed, thereby promoting tumor progression. The stiffness of ECM in TME also affects the efficacy of radiotherapy. Using a 3D culture model of Huh7 human liver cancer cells with different stiffness levels, Lu et al. found that LOX plays a significant role in tumor radioresistance by promoting ECM stiffness and matrix remodeling [107]. Their results suggested that inhibiting LOX activity can reduce matrix stiffness, enhance IR-induced DNA damage responses in cirrhotic matrices, and reverse radioresistance. Notably, this inhibitory effect is limited in normal liver matrices.

Through bioinformatics and mass spectrometry-based proteomics, LOXL2 has been implicated in radioresistance in prostate cancer and LOXL2 is persistently upregulated in castration-resistant prostate cancer (CRPC) cells. Knockdown of LOXL2 significantly enhanced the radiosensitivity, which may be attributed to the reversal of the EMT process [108]. Epithelial morphology changes and the reappearance of cell-cell adhesion were observed in vitro. In the xenograft mouse model, increased expression of the EMT biomarker E-cadherin was noted following LOXL2 knockdown, and salvage experiment could restore radioresistance. Samaržija et al. analyzed different sublines of CRPC cell line DU145: DU145 parental (DU145 P) and radioresistant (DU145 RR) cells [109] and demonstrated that LOXL2 was upregulated in both cell sublines. However, LOXL2 knockdown did not significantly affect the radiosensitivity of DU145 RR cells, and instead induced radioresistance in DU145 P cells. These inconsistent results suggest that additional factors and studies should be considered to evaluate whether LOXL2 is an effective target for overcoming treatment resistance in CRPC cells. Additionally, in the IR-induced senescence process, LOXL1 protein expression was found to be downregulated in human umbilical vein endothelial cells (HUVECs), which may lead to the loss of venous elasticity [110].

### Radiation-induced fibrosis

Radiation-induced fibrosis (RIF) is a late adverse effect of radiotherapy, which may occur in any tissue or organ within the treatment field. It is characterized by impaired wound healing and persistent inflammation, driven by cumulative radiation-induced damage to both cells and extracellular components, ultimately resulting in disordered tissue remodeling and abnormal collagen deposition in the ECM [111]. Chronic inflammation induced by IR is a key extracellular factor contributing to fibrosis. The migration and infiltration of numerous inflammatory cells lead to the secretion of inflammatory cytokines and the activation of myofibroblasts. Among these cells, macrophages are the most representative. Choi et al. recently reported that radiation-induced tissue injury promotes the infiltration and M2-like polarization of monocyte-derived macrophages, thereby increasing TGF-β1 production and activating SRC family kinase (SFK) signaling in epithelial cells and fibroblasts, which drives fibrotic remodeling [112]. In addition, cellular senescence and the activation of extracellular profibrotic signaling molecules, especially TGF-β-centered signaling pathways, further contribute to the development of RIF. In parallel, intracellular mechanisms include radiation-induced DNA damage, oxidative stress, metabolic reprogramming, and proteostasis dysregulation. Epigenetic regulatory networks involving DNA methylation, histone modifications, and non-coding RNA-mediated mechanisms have also attracted increasing attention [113]. Overall, these established mechanisms converge on the deposition, maturation, and stiffening of the ECM, suggesting that enzymes responsible for matrix stabilization may play an important role in the persistence and irreversibility of RIF.

Thoracic malignancies, particularly breast and lung cancers, are among the most common cancers in clinical practice [114]. The incidence of radiation-induced lung fibrosis (RILF) following thoracic radiotherapy ranges from approximately 9% to 30% [115]. In the mouse model of RILF, Chung et al. demonstrated that TGF-α, a ligand of epidermal growth factor receptor (EGFR), promotes LOX expression and activity in fibroblasts by activating the PI3K/Akt/mTOR signaling pathway, thereby enhancing collagen deposition [116]. This effect is dependent on sustained TGF-α expression rather than TGF-β involvement. In addition, the expression of LOX is regulated by intracellular signaling proteins and transcription factors activated in response to irradiation, predominantly through the IκB kinase β (IKKβ)/nuclear factor kappa B (NF-κB) signaling pathway. Inhibition of IKKβ or NF-κB can downregulate LOX transcription and synthesis in irradiated fibroblasts, thereby alleviating RILF [117].

**Fig. 4:**
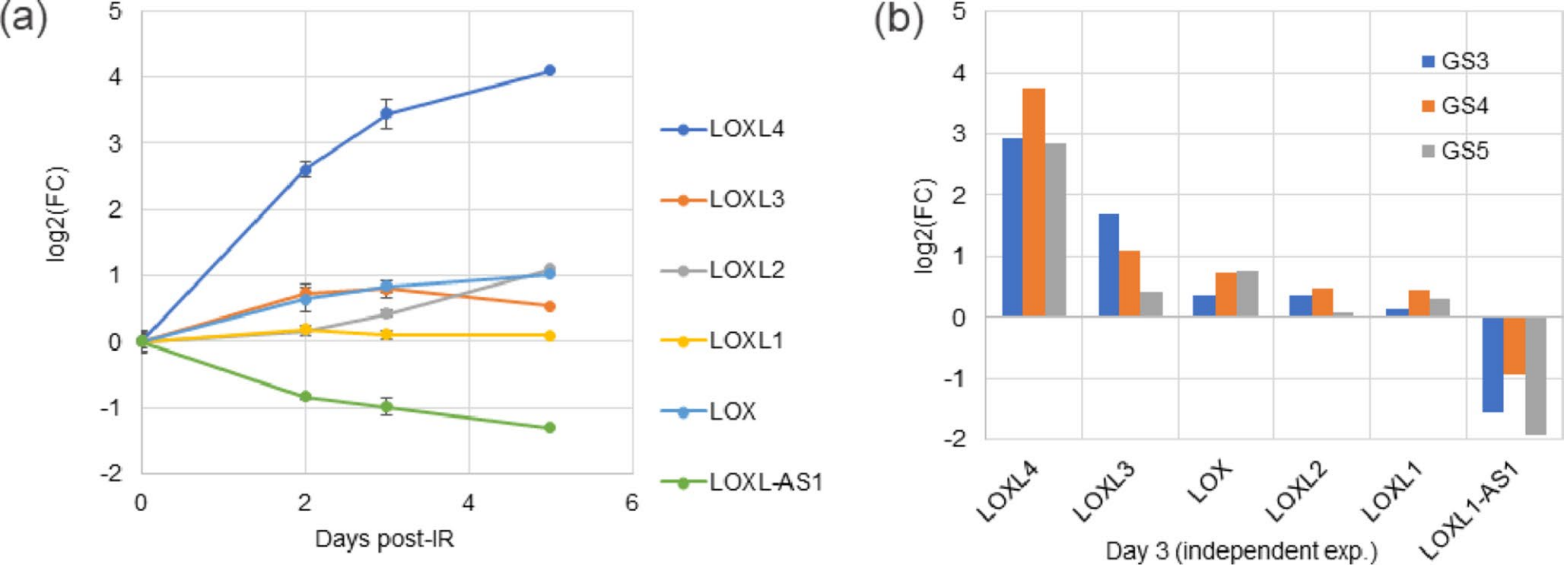
Radiation-induced expression of LOX family mRNA in early-passage skin fibroblasts. (a) log2(FC) (fold change) on day 2, 3, and 5 after a single dose of 4 Gy of 6 MV X-rays. Mean values and ranges of two independent experiments with strain GS4 are shown. Most of the error bars are smaller than the symbols. (b) log2(FC) on day 3, additional, independent experiments with strains GS3, GS4 and GS5. Affymetrix Human Genome U133 plus 2.0 microarrays were used. For experimental details and qPCR validation of LOXL4 expression kinetics over day 1–6, see Herskind et al. [119]

Skin is another commonly affected organ in RIF [111], especially after RT for breast or head-and-neck tumors. Recent LOX-related research on skin mainly focused on its role under ultraviolet radiation (UVR) exposure [118]. However, our group has investigated changes in gene expression profiles during ionizing radiation-induced premature differentiation of early-passage, human dermal fibroblasts. Transcription of LOXL4 was strongly upregulated in these cells following irradiation, with qPCR results showing an up to 20-fold increase [119]. This would seem consistent with the previously mentioned role of LOXL4 in pathological collagen crosslinking and skin fibrosis [46]. Further analysis of the gene expression microarrays revealed significant upregulation of LOX but not LOXL1, whereas LOXL1-AS1 antisense was significantly downregulated (Fig. 4), suggesting epigenetic upregulation of LOXL1 at the post-transcriptional level. LOXL3 was upregulated two-to-three-fold in two fibroblast strains (GS3, GS4) which - given the quantitative reproducibility of the other genes in these five independent experiments, including three repeats with GS4 - may indicate some degree of variation in expression of LOXL3 (and possibly LOXL2) between individual donors.

Taken together, these findings suggest that LOXL4, LOX and LOXL1 may play a critical role in radiation-induced skin fibrosis by modulating not only ECM accumulation but also its mechanical properties, making these LOX family members potential target for further research and antifibrotic intervention. Moreover, a recent study revealed a tissue-specific mechanism underlying RIF in prostate stroma. KDM3B, a histone demethylase, was found to regulate LOX expression at the post-transcriptional level in prostate stromal cells through N^6^-methyladenosine (m^6^A) modification of its transcript, thereby contributing to collagen crosslinking and tissue stiffening [120]. By inspecting the RNA-seq datasets from the epigenetic study by Bian et al. on radiotherapy-induced memory in dermal fibroblasts [121], we further noted a dynamic and isoform-specific regulation of LOX family members during human wound healing, with different LOX/LOXL genes showing opposite directions of change at the same time point.

## Therapeutic approaches targeting LOX and LOXLs

The first-generation inhibitor β-aminopropionitrile (BAPN) irreversibly inhibits the activity of LOX and LOXL proteins by competitively binding to their active sites [122]. It is widely used for investigating the biological functions of the LOX family members in various experimental settings but owing to toxicity in blood vessels and bones, and potential off-target effects, BAPN is not considered suitable for clinical translation [12]. However, other inhibitors with less side effects and higher bioavailability have been developed, including selective inhibitors and novel small-molecule pan-LOX inhibitors (Table 2).

**Table 2:** Inhibitors targeting the lysyl oxidase family and their current status

Compound	Study Start		Clinical Trial ID		Phase	Disease(s)	Treatment	Primary Outcome Measures
BAPN		n/a		n/a	n/a	Fibrotic diseases	pan-LOX inhibitor	n/a
Simtuzumab (Completed)		2011		NCT01452308	IIa	liver fibrosis	LOXL2 monoclonal antibody	Safety and tolerability
		2011		NCT01369498	II	Primary, post polycythemia vera or post essential thrombocythemia myelofibrosis		Rate of clinical response
		2011		NCT01472198	II	Metastatic pancreatic adenocarcinoma		PFS
		2012		NCT01707472	IIa	HIV- and/or hepatitis C- infected subjects with liver fibrosis		Safety and tolerability
		2013		NCT01672853	IIb	liver fibrosis with PSC		Change in MQC on Liver Biopsy
		2015		NCT02466516	II	NASH and fibrosis stages F2-F3		Safety and tolerability
Simtuzumab (Terminated)		2011		NCT01479465	II	Colorectal adenocarcinoma	LOXL2 monoclonal antibody	PFS
		2012		NCT01759511	II	IPF		Safety and tolerability
		2012		NCT01672866	IIb	Advanced liver fibrosis but not cirrhosis secondary to NASH		Change in MQC, EFS
		2012		NCT01672879	IIb	Cirrhosis due to NASH		Change in HVPG, EFS
		2013		NCT01769196	II	IPF		PFS
PXS-S1A		n/a		n/a	pre-clinical	Breast cancer	LOX/LOXL2 inhibitor	n/a
PXS-S2A		n/a		n/a	pre-clinical	Breast cancer	LOXL2 inhibitor	n/a
Compound 20		n/a		n/a	lead identification	n/a	LOXL2 inhibitor	n/a
PAT-1251 (GB2064)		2021		NCT04679870	IIa	Myelofibrosis	LOXL2 inhibitor	Safety and tolerability
SNT-5382 (PXS-5382A)		2017		ACTRN12617001564347	I	Safety evaluation, with potential future application in heart failure	LOXL2 inhibitor	Safety and tolerability
CCT365623		n/a		n/a	pre-clinical	Spontaneous breast tumor lung metastasis	LOX/LOXL2 inhibitor	n/a
PXS-5153A		n/a		n/a	pre-clinical	Liver fibrosis Heart fibrosis	LOXL2/LOXL3 inhibitor	n/a
PXS-5120A/ PXS-5129A		n/a		n/a	pre-clinical	Liver fibrosis Lung fibrosis	LOXL2/LOXL3 inhibitor	n/a
PXS-5338		2017		ACTRN12617001444370	I	NASH and other fibrosis-based diseases	LOXL2/LOXL3 inhibitor	Safety and tolerability
PXS-6302		2021		ACTRN12621000322831	I	Acute and established scar	pan-LOX inhibitor	Safety and tolerability
PXS-5505		2021		NCT04676529	I/IIa	Advanced myelofibrosis	pan-LOX inhibitor	Safety and tolerability

n/a: not available, PFS: progression free survival, PSC: primary sclerosing cholangitis, NASH: non-alcoholic steatohepatitis, IPF: idiopathic pulmonary fibrosis, HVPG: hepatic venous pressure gradient, EFS: event free survival, MQC: mean quantitative change

LOXL2 is the most widely explored and the first member of the LOX family to have a precursor crystal structure [123]. Simtuzumab is an inhibitory humanized monoclonal antibody targeting LOXL2. Its rodent equivalent is AB0023, which can bind to the SRCR-4 domain of human LOXL2 and has shown efficacy in various rodent models of tumors and fibrotic diseases. However, clinical trials have shown no clinical improvement in reducing fibrosis in multiple organs, leading to the termination of its development [124]. Small molecule LOXL2 inhibitors include PXS-S1A and PXS-S2A based on halogenated allylamine, PAT-1251 (also known as GB2064) which has entered clinical trial stage, and SNT-5382 which has recently shown good safety profile in phase I clinical trial and potential to reduce fibrosis [14, 125]. They also work by binding to the active site of the enzyme but offer better chemical tractability and enhanced specificity. Furthermore, LOX/LOXL2 inhibitors with aminomethylthiofene (AMT) and aminomethylthiazole (AMTz) scaffolds, and potent LOXL2/LOXL3 inhibitors such as PXS-5153A, PXS-5120A, and PXS-5338, are under development [14, 82]. These dual inhibitors improve therapeutic efficacy through multi-target intervention, while also helping to clarify the role of LOX family in disease progression.

With a deeper understanding of the mechanisms of LOX family members, it seems likely that compensatory interactions between isoenzymes and the complex involvement at different disease stages may be the cause of previous clinical failures [124]. Pan-LOX inhibitors become an attractive option. PXS-6302 is an irreversible pan-LOX inhibitor with higher targeting capability and permeability than BAPN. It is applied locally after skin injury and intended to ameliorate scar formation. The PXS-6302-related clinical trial was registered and initiated in 2021. By 2025, the results of the phase I trial showed that topical application was well tolerated and modulated ECM-related scar parameters, supporting further evaluation in a phase II trial [126]. PXS-5505 is another novel irreversible pan-LOX inhibitor but for systemic treatment. It has the advantages of high oral bioavailability, high specificity for the LOX family, and good tolerability. PXS-5505 has demonstrated safety and significant efficacy in both various preclinical models (including solid tumors, myeloid malignancies, and multi-organ fibrosis) and a phase I/IIa clinical trial for advanced myelofibrosis [127], making it a potential candidate for antifibrotic therapy as well as for combination with chemotherapy in cancer treatment.

In addition, recent advances have expanded the application of LOX/LOXL targets as biomarkers and imaging probes. LOXL2-specific SPECT imaging has been developed for IPF [128], while LOX-responsive collagen peptides can provide real-time spatiotemporal information on collagen formation and remodeling during tissue repair [129]. LOX-engineered nanoparticles also demonstrate good synergistic effects with chemotherapy in both *in vitro* and *in vivo* models of TNBC [130].

## Conclusions and perspectives

The LOX family members are vital for maintaining ECM integrity, yet are also directly involved in the pathogenesis of fibrotic diseases and tumor progression, as evidenced by their frequent overexpression in the fibrosis of normal tissues and in the tumor stroma. In addition to directly affecting ECM stiffness, they also play important roles in signaling pathways regulating ECM deposition and cell phenotype. Their contribution to ECM remodeling and radiation-related effects emphasizes their potential as therapeutic targets. Further research should focus on elucidating the molecular mechanisms of LOX/LOXL functions (particularly after irradiation), clarifying the protein crystal structures for rational drug design, and evaluating the clinical benefits. Of note, careful consideration must be given to balancing the physiological roles of LOX/LOXL with its therapeutic inhibition.

## Data Availability

The gene expression data shown in this review were obtained as part of a previous study [155]. The raw and normalized data are deposited in the Gene Expression Omnibus (GEO) database (http://www.ncbi.nlm.nih.gov/geo/; accession number GSE147733).

## Abbreviations

α-SMA: α-smooth muscle actin
AP-1: Activator protein 1
BAPN: β-aminopropionitrile
BLM: Bleomycin
BMDC: Bone marrow-derived cell
BMP1: Bone morphogenetic protein 1
CAF: Cancer-associated fibroblast
CCl_4_: Carbon tetrachloride
CKD: Chronic kidney disease
COPD: Chronic obstructive pulmonary disease
CRL: Cytokine receptor-like
CRPC: Castration-resistant prostate cancer
CTGF: Connective tissue growth factor
ECM: Extracellular matrix
EGFR: Epidermal growth factor receptor
ELISA: Enzyme-linked immunosorbent assay
EMT: Epithelial-mesenchymal transition
EndMT: Endothelial-mesenchymal transition
ER: Endoplasmic reticulum
FAK: Focal adhesion kinase
FMT: Fibroblast-to-myofibroblast transition
GC: Gastric cancer
HIF-1α: Hypoxia-inducible factor-1α
H₂O₂: Hydrogen peroxide
HPC: Hepatic progenitor cell
HSC: Hepatic stellate cell
HUVEC: Human umbilical vein endothelial cell
IGF1: Insulin-like growth factor 1
IKKβ: IκB kinase β
IL: Interleukin
ILD: Interstitial lung disease
IMRT: Intensity-modulated radiotherapy
IPF: Idiopathic pulmonary fibrosis
IR: Ionizing radiation
LOX: Lysyl oxidase
LOXL: Lysyl oxidase-like enzymes
LTQ: Lysine tyrosylquinone
MMP: Matrix metalloproteinase
MRI: Magnetic resonance imaging
mTOR: Mechanistic target of rapamycin
NAFLD: Non-alcoholic fatty liver disease
NF-κB: Nuclear factor kappa B
PD-L1: Programmed death-ligand 1
PDGF: Platelet-derived growth factor
PKB: Protein kinase B
PMF: Primary myelofibrosis
PPF: Progressive pulmonary fibrosis
RIF: Radiation induced fibrosis
RILF: Radiation-induced lung fibrosis
ROS: Reactive oxygen species
RT: Radiotherapy
SBRT: Stereotactic radiotherapy
SRCR: Scavenger receptor cysteine-rich
SSc: Systemic sclerosis
TAM: Tumor-associated macrophage
TGF-β: Transforming growth factor β
TME: Tumor microenvironment
TNBC: Triple negative breast cancer
UVR: Ultraviolet radiation
VEGF: Vascular endothelial growth factor
YAP: Yes-associated protein
